# Two Novel Cycloartane-Type Triterpenes from *Trichilia casaretti* C. DC. (Meliaceae)

**DOI:** 10.3390/molecules23040949

**Published:** 2018-04-19

**Authors:** Ivo Jose Curcino Vieira, Elaine Rodrigues Figueiredo, Milena Gonçalves Curcino Vieira, Almir Ribeiro de Carvalho Junior, Michel de Souza Passos, Samyra Imad da Silva Boeno, Otoniel Aquino Azevedo, Raimundo Braz-Filho

**Affiliations:** 1 Setor de Química de Produtos Naturais, Universidade Estadual do Norte Fluminense Darcy Ribeiro, Campos dos Goytacazes 28013-602, Rio de Janeiro, Brazil; Almir@uenf.br (A.R.d.C.J.); michel.s.p_35@hotmail.com (M.d.S.P.); smr.imad@gmail.com (S.I.d.S.B.); braz@uenf.br (R.B.-F.); 2 Instituto Federal Fluminense, Campus Bom Jesus do Itabapoana, Avenida Dario Vieira Borges, 235, Parque do Trevo, Bom Jesus do Itabapoana 28360-000, Rio de Janeiro, Brazil; elaine.figueiredo@iff.edu.br; 3 Faculdade de Medicina de Campos, Avenida Alberto Torres, 217, Centro Campos dos Goytacazes 28035-581, Rio de Janeiro, Brazil; milena.uff@gmail.com; 4 Centro Universitário São Camilo, Campus I, Rua São Camilo de Léllis 01, Cachoeiro de Itapemirim 29304-910, Espirito Santo, Brazil; otoazevedo@gmail.com; 5 Departamento de Química, Universidade Federal Rural do Rio de Janeiro, CP 74541, Seropédica 23890-000, Rio de Janeiro, Brazil

**Keywords:** *Trichilia casaretti*, Meliaceae, triterpenes, cycloartane-type

## Abstract

Two new triterpenes cycloartane-type, named 24-methylencycloartan-12-oxo-3β,22α-diol and trichiliol, were isolated from the leaves of *Trichilia casaretti* C. DC. together with three known triterpenes—24-methylencycloart-3β,22-diol, 22,25-dihydrocycloart-23(*E*)-en-3β-ol, and 22(*R*)-hydroxycycloart-24-en-3-ol. These compounds were characterized on the basis of their spectral data, mainly 1D (^1^H and ^13^C) and 2D NMR (^1^H-^1^H-COSY, ^1^H-^1^H-NOESY, HMQC, HSQC, and HMBC), and mass spectra (EI-MS and HR-ESI-MS), also involving comparison with data from the literature.

## 1. Introduction

The Meliaceae family has attracted such a great interest among phytochemists because of its relatively complex and diverse chemical structures and its biological activity, mainly against insects [1,2,3,4]. The *Trichilia* genus (Meliaceae) comprises about 230 species distributed throughout tropical America, which are recognized for their significant economic importance and high commercial value. Phytochemical studies revealed that this genus is a potential source of terpenoids, including triterpenes, limonoids, steroids and other terpenes derivatives [3,4,5,6]. Species of this genus have been also studied for their insecticidal activities and their isolated compounds revealed complex and interesting structures including various limonoids [5,7,8]. The isolation and structural elucidation of two novel sesquiterpenes from the stems of *T. casaretti* collected in Espírito Santo State, Brazil, were reported by Vieira et al. in 2010 [4].

**Figure 1:**
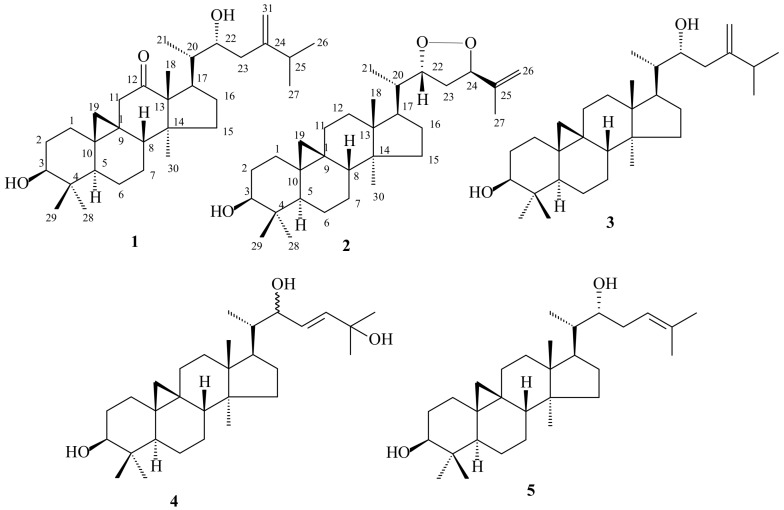
Triterpenes isolated from *Trichilia casaretti.*

In the present article, we describe an investigation of methanol extract from leaves of a *T. casaretti* specimen, which allowed the characterization of five triterpenes, including two novel cycloartane-type triterpenes named 24-methylencycloartane-12-oxo-3β,22α-diol (**1**) and trichiliol (**2**). The known triterpenes were identified as 24-methylencycloartane-3β,22-diol (**3**), cycloartane-22(*E*)-en-3β,22,25-triol (**4**), and 22(*R*)-hydroxycycloart-24-en-3-ol (**5**). Their structures (Figure 1) were established by spectrometric techniques, mainly 1D and 2D NMR, and HR-ESI-MS, and comparison with literature data.

## 2. Results and Discussion

Fractionation of the MeOH extract of the leaves from *T. casaretti* by classical chromatographic methods resulted in the isolation of five cycloartane-type triterpenes **1**–**5** whose structures are shown in Figure 1. The two novel cycloartane-type triterpenes named 24-methylencycloartan-12-oxo-3β,22α-diol (**1**) and trichiliol (**2**), along with three known triterpenes, cycloartane-type 24-methylencycloart-3β,22-diol (**3**) [9,10], 22,25-dihydrocycloart-23(*E*)-en-3β-ol (**4**) [9,11] and 22(*R*)-hydroxycycloart-24-en-3-ol (**5**) [12] were characterized on the basis of ^1^H and ^13^C-NMR spectral data, especially 2D NMR and mass spectral data, besides comparison with values described in the cited literature.

The new triterpene named 24-methylencycloartan-12-oxo-3β,22α-diol(**1**) was isolated as yellow oil, ${\left[\alpha \right]}_{D}^{23}$ = +27.3 (CHCl_3_, *c* 0.02). The analysis of the {^1^H}- and ^13^C-APT NMR spectra (Table 1) revealed signals corresponding to 31 C-atoms, including seven quaternary C (five sp^3^ and two sp^2^, one attributed to methylidene at δ_C_ 153.5 (C-24) and one C=O group at δ_C_ 216.0 (C-12)), seven CH (all sp^3^, including two linked to *O* atom at δ_C_ 78.8 (CH-3) and 70.3 (CH-22), nine methylene CH_2_ (including one sp^2^ at δ_C_ 109.8 (CH_2_-31)) and seven methyl groups CH_3_, allowing to deduce the expanded partial formula C_6_(C=O)CH_5_(OCH)_2_(CH_2_)_10_(CH_3_)_7_ = C_31_H_48_O_3_ or C_6_(C=O)CH_5_(HOCH)_2_(CH_2_)_10_(CH_3_)_7_ = C_31_H_50_O_3_ based in the presence of two hydroxyl groups.

The HR-ESI-MS spectrum of **1** showed peak corresponding to the quasi molecular ion ([M + H]^+^) at *m*/*z* 471.3475, that in combination with ^1^H-NMR (1D and 2D) and comparative analysis of the {^1^H}- and ^13^C-APT NMR spectra (Table 1) enabled us to propose the molecular formula C_31_H_50_O_3_ (seven degrees of unsaturation) compatible with a cycloartane-type 24-methylencycloart-3β,22-diol (**3**) skeleton [9]. The only difference regarding compounds **1** and **3** consists of a carbonyl group at C-12.

The signal at δ_C_ 216.0 in the ^13^C-APT NMR spectrum suggested the presence of a carbonyl group in the triterpene cycloartane **1**. The location of the carbonyl group at C-12 was supported in the HMBC spectrum by the correlation (^3^*J*_HC_) between C-12 (δ_C_ 216.0) and the hydrogens of the methyl group 3H-18 at δ_H_ 0.90. Thus, the new triterpene cycloartane-type was characterized as 24-methylidencycloartan-12-oxo-3β,22-diol (**1**).

Compound **2** was isolated as a yellow oil, ${\left[\alpha \right]}_{D}^{23}$= +48.0 (CHCl_3_, *c* 0.001). The analysis of the {^1^H}- and ^13^C-NMR spectra (Table 2) revealed signals corresponding to 30 carbon atoms, showing similarity with the triterpene cycloartane **1**. Comparison of the ^13^C-NMR data of compounds **1** and **2** revealed the absence of carbonyl signal in **2**, and additional differences can be justified through modifications involving only the side chain (Table 1 and Table 2).

The ^13^C-NMR spectrum of **2** with the presence of 30 carbon signals revealed additional carbon signals corresponding to remaining carbon atoms (C)(CH)(OCH)_2_(CH_2_)_2_(CH_3_)_2_ = C_8_H_13_O_2_ of the side chain, different of the cycloartane triterpene **1** with signals representing 31 carbon atoms including (C)(CH)_2_(HOCH)(CH_2_)_2_(CH_3_)_3_ = C_9_H_17_O of the side chain.

The ^13^C-NMR spectrum involving the side chain of **2** revealed signals representing only two methyl groups when compared with those of **1** with three, one corresponding to CH_3_-21 at δ_C_ 19.5 correlated in the HSQC with the doublet (*J* = 6.3 Hz) signal at δ_H_ 1.22 (3H-21) and one attached to sp^2^ carbon (CH_3_-27, δ_H_ 2.01\δ_C_ 18.5), two sp^2^ carbon atoms at δ_C_ 147.8 (C-25) and 112.5 (CH_2_-26), featuring a methylidene group =CH_2_ confirmed by correlations (^1^*J*_HC_) between δ_C_ 112.48 (CH_2_-26) with two broad singlets at δ_H_ 5.32 (Ha-26) and 5.10 (Hb-26) observed in the HSQC spectrum.

The location of the methylidene group at the C-25 was confirmed through the ^2^*J*_HC_ correlations between C-25 (δ_C_ 147.8) and 3H-27 (δ_H_ 2.01) and C-26 (δ_C_ 112.5) with 3H-27 (δ_H_ 2.01). The presence of a isopropenyl group (isopropyl in **1**) attached to CH-24 (δ_C_ 86.6\δ_H_ 5.32, *br s*) was deduced by HSQC and HMBC correlations between CH-24 and 3H-27 [(δ_H_ 2.01, *br s*\(δ_C_ 18.5, ^3^*J*_HC_)]. It was also possible to observe the interaction between CH-22 (δ_C_ 69.87.6\(δ_H_ 4.55, *br d*, *J* = 10.0 Hz) and 3H-21 (δ_H_ 1.22, *d*, *J* = 6.3 Hz\(δ_C_ 12.8, ^3^*J*_HC_). These data allowed to postulate the presence of oxygen atoms linked to carbon 22 (HC-O-22: δ_H_ 4.55\69.7) and 24 (HC-O-24 δ_H_ 5.32\86.6), in agreement with the ^1^H-^1^H-COSY spectrum which showed correlations between the methylene hydrogens 2H-23 (δ_H_ 2.10 and 1.90\δ_C_ 34.5) and both hydrogen atoms H-22 (δ_H_ 4.55) and H-24 at δ_H_ 5.32 (chemical shift justified by location in position allylic and carbinolic). Other heteronuclear correlations observed in the HMBC spectrum of **2** were shown in Table 2.

The endoperoxide function in the side chain was also supported by mass spectra of low and high resolution demanding an additional ring to suit the seven degrees of unsaturation. The HRESI-MS spectrum of **2** (Scheme S1, Supplementary Materials) showed peak corresponding to the protonated molecular ion [M + H]^+^ at *m*/*z* 457.3502 which together with the NMR spectrum of ^13^C-DEPT 135° NMR allowed to propose the molecular formula C_30_H_48_O_3_ compatible with a cycloartane-type skeleton and one heterocyclic involving the peroxide function.

**Figure 2:**
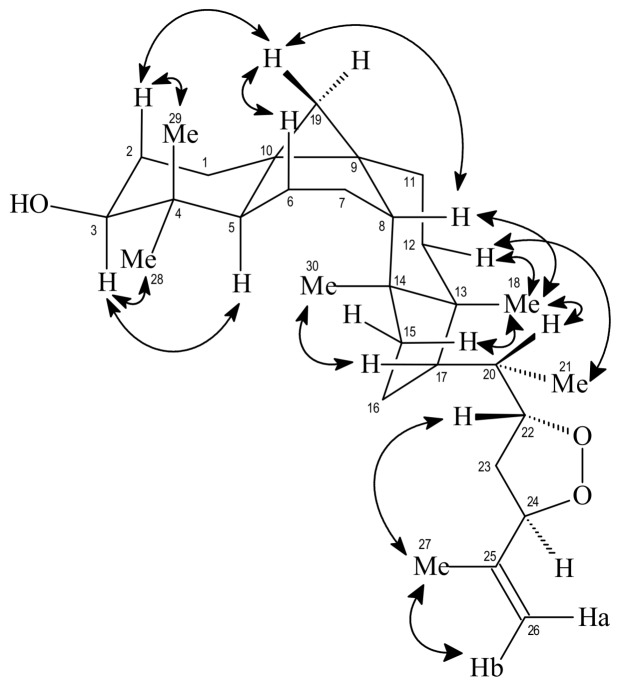
Selected Nuclear Overhauser Effect (NOE) correlations and relative stereochemistry for compound **2**. Arrows denote the main NOE correlations.

The relative stereochemistry indicated in **2** was deduced by the spatial dipolar interaction observed in the ^1^H-^1^H-NOESY spectrum between 3H-30 and H-17 indicating H-17α, interaction of 3H-18 with H-20 and 3H-21with H-12 equatorial suggesting H-20α and CH_3_-21α, respectively. Other spatial dipolar interaction was shown in Figure 2. These data allowed to identify compound **2** as the new triterpene 22,24-peroxidecycloart-25-en-3β-ol (**2**), named trichiliol.

## 3. Material and Methods

### 3.1. General Methods

Measures of optic rotation were obtained on a Perkin Elmer 343 digital polarimeter. Melting point was obtained on a Microquímica MQRPF. EI-MS (low resolution) mass spectra were obtained on Shimadzu QP5050A mass spectrometer. HRESI-MS (high resolution) mass spectra were obtained by using a ESI-IT-OF-MS SHIMADZU mass spectrometer, using the positive ion mode of analysis. Chromatographic purifications were carried out over silica gel 60 (70–230 mesh). Silica gel 60 F_254_ was used in thin layer chromatography analysis. ^1^H and ^13^C-NMR spectra were measured on a Bruker model DRX-500 and Jeol model Eclipse-400 spectrometers, equipped with inverse probes and field gradient, operating at 500 (^1^H) and 125 (^13^C) and 400 (^1^H) and 100 (^13^C) MHz. CDCl_3_ and pyridine-*d*_5_ were used as solvents and TMS as internal reference. Chemical shifts are given in the δ scale (ppm) and coupling constants *J* in Hz. One dimensional (1D) ^1^H and ^13^C-NMR spectra were acquired under standard conditions by using a direct detection 5 mm ^1^H/^13^C dual probe. Standard pulse sequences were used for two dimensional spectra by using a multinuclear inverse detection 5 mm probe with field gradient.

### 3.2. Plant Material

The leaves of *Trichilia casaretti* C. DC. were collected at Vale Cia, Linhares City, Espirito Santo State, Brazil, and identified by Domingos Folly. A voucher specimen (CVRD-449) was deposited at the Vale Cia of Vale herbarium, Vale Cia, Linhares City, Espirito Santo State, Brazil.

### 3.3. Extract and Isolation

Air dried and powdered leaves (920 g) from *Trichilia casaretti* C. DC. were extracted with methanol at room temperature, yielding 15.0 g of crude methanol extract. The methanol extract was submitted to liquid-liquid partition (CH_2_Cl_2_:H_2_O, 3:1, *v*/*v*). The CH_2_Cl_2_ fraction (10.1 g) was submitted to liquid-liquid partition (MeOH:Hexane, 1:1, *v*/*v*). The methanol fraction (8.0 g) was chromatographed over a silica gel column with a gradient of ethyl acetate/hexane, affording eight fractions. Fraction 1 (19.3 mg) was similarly rechromatographed, yielding triterpene **5** (8.0 mg). Fraction 5 (518 mg) was analogously rechromatographed, affording eleven fractions. Fraction 5.7 (66.8 mg) was similarly chromatographed, affording triterpene **4** (4.0 mg). Fraction 7 (6.736 mg) was chromatographed affording fifteen fractions. The fraction 7.5 (1.300 mg) was rechromatographed affording fourteen fractions. The triterpene **2** (10 mg) was obtained from the fractions 7.5.3 (45 mg) and 7.5.4 (100 mg). The triterpene **1** (25 mg) was obtained from the fraction 7.6 (500 mg). The fraction 7.9 (163 mg) was chromatographed affording fifteen fractions. The fraction 7.9.2 (50.3 mg) yielded triterpene **3** (11 mg).

## Figures and Tables

**Table 1:** ^13^C and ^1^H-NMR data of triterpene **1** and comparison with triterpene **3** (CDCl_3_), δ in ppm, coupling constants (*J* in Hz, in parenthesis) *.

C	1				3
		HMQC (H→C)	HMBC (C→H)		
	δ (C)	δ (H)	^2^ *J*	^3^ *J*	δ (C)
1	32.8	1.65–1.55 (*m*, 2H)		2H-19	32.0
2	32.0	1.78–1.80 (*m*, 1H) 1.62–1.55 (*m*, 1H)			30.4
3	78.8	3.28 (*dd*, 11.1; 4.1, 1H)		3H-28, 3H-29	78.8
4	40.7	-	3H-28, 3H-29		40.8
5	48.0	1.30–1.25 (*m*, 2H)		3H-28, 3H-29	47.1
6	21.6	1.58–1.62 (*m*, 1H) 0.78–0.80 (*m*, 1H)			21.1
7	25.4	1.28–1.30 (*m*, 1H) 1.10–1.16 (*m*, 1H)			27.2
8	47.1	1.30 (*br s*, 1H)		3H-30	48.0
9	19.5	-			20.0
10	29.9	-			26.1
11	27.2	n.d.		2H-19	26.1
12	218.0	-		3H-18	35.7
13	49.0	-	3H-18	3H-30	45.8
14	48.0	-	3H-30	3H-18	48.4
15	45.7	1.30–1.34 (*m*, 2H)		3H-30	32.9
16	25.4	2.01–2.05 (*m*, 1H) 1.30–1.34 (*m*, 1H)			26.4
17	49.0	1.49–1.53 (*m*, 1H)		3H-18, 3H-21	49.0
18	12.1	0.91 (*s*, 3H)		H-12, H-17	18.0
19	30.3	0.56 (*d*, 4.1, 1H) 0.35 (*d*, 4.1, 1H)		2H-1	29.9
20	40.9	1.88–1.80 (*m*, 1H)	3H-21		40.5
21	19.5	0.90 (*d*, 5.8, 3H)	H-20	H-17, H-22	12.0
22	70.3	3.78 (*br d*, 10.6, 1H)			70.4
23	36.1	2.24 (*d*, 13.5, 1H) 1.95 (*dd,* 13.5; 11.2, 1H)	H-22	2H-31	36.1
24	153.5	-	2H-23, 2H-31	3H-26, 3H-27	153.6
25	33.1	2.20–2.24 (*m*, 1H)			33.2
26	22.3	1.05 (*d*, 7.0, 3H)			22.3
27	21.6	1.08 (*d*, 7.0, 3H)			21.6
28	25.4	0.97 (*s*, 3H)		H-3, H-5, 3H-29	19.5 ^a^
29	14.2	0.81 (*s*, 3H)		H-3, H-5, 3H-28	14.0
30	18.0	1.01 (*s*, 3H)		H-8, 2H-15	25.4 ^a^
31	109.8	4.93 (*s*, 1H)/4.84 (*s*, 1H)		2H-23	109.7

* Number of hydrogens bound to carbon atoms deduced by comparative analysis of {^1^H}- and APT-^13^C-NMR spectra. Chemical shifts and coupling constants (*J*) obtained of 1D ^1^H-NMR spectrum. Superimposed ^1^H signals are described without multiplicity and approximated chemical shifts deduced by HMQC, HMBC and ^1^H-^1^H-COSY spectra. Assignments determined by a combination of 1D and 2D (^1^H-^1^H-COSY, HSQC and HMBC) NMR experiments. ^a^ Chemical shifts with same letter cam be exchanged. n.d. = not detected.

**Table 2:** ^13^C- and ^1^H-NMR data of triterpene **2** (pyridine-*d*_5_), δ in ppm, coupling constants (*J* in Hz, in parenthesis) *.

C	2			
		HSQC (H→C)	HMBC (C→H)	
	δ (C)	δ (H)	^2^ *J*	^3^ *J*
1	32.8	1.50–1.53 (*m*, 1H) 1.25–1.26 (*m*, 1H)		2H (19)
2	31.6	2.00–2.01 (*m*, 1H) 1.94–1.98 (*m*, 1H)		
3	78.3	3.57 (*br d*, 9.0, 1H)		3H-28, 3H-29
4	41.5	-	3H-28, 3H-29	
5	47.9	1.33 (*m*, 1H)	2H-6	3H-28, 3H-29
6	21.8	1.60–1.65 (*m*, 1H) 0.77–0.82 (*m*, 1H)	H-5	
7	26.8	1.12–1.05 (*m*, 2H)	H-8	
8	48.6	1.46–1.50 (*m*, 1H)	2H-7	3H-30
9	20.3	-	2H-19	
10	26.9	-	H-5, 2H-19	
11	27.0	2.00–2.10 (*m*, 1H) 1.25–1.30 (*m*, 1H)		2H-19
12	33.4	1.60–1.72 (*m*, 2H)		3H-18
13	46.2	-	3H-18-	3H-30
14	48.9	-	3H-30	3H-18
15	36.3	2.10–2.20 (*m*, 1H) 1.20–1.30 (*m*, 1H)		3H-30
16	28.0	2.22–2.38 (*m*, 1H) 1.90–2.00 (*m*, 1H)		
17	49.9	1.80 (*br q*, 9.0, 1H)		3H-18, 3H-21
18	18.7	1.03 (*s*, 3H)		H-17
19	30.5	0.54 (*br s*, 1H) 0.33 (*br s*, 1H)		2H-1, 2H-11
20	43,6	2.05–2.25 (*m*)	3H-21-	
21	12.8	1.22 (*d*, 6.3, 3H)	H-20	H-17, H-22
22	69.7	4.55 (*br d*, 10.0, 1H)		3H-21
23	34.5	2.20–2.30 (*m*, 1H) 1.85–1.90 (*m*, 1H)		
24	86.6	5.32 (*br s*, 1H)		3H-27
25	147.8	-	3H-27	
26	112.5	5.32 (*br s*, 1H) 5.10 (*br s*, 1H)		3H-27
27	18.5	2.01 (*br s*, 3H)	H-25	H-24, 2H-26
28	26.6	1.25 (*s*, 3H)		H-3, H-5, 3H-29
29	15.2	1.22 (*s*, 3H)		H-3, H-5, 3H-28
30	20.1	0.89 (*s*, 3H)		H-8, 2H-15

* Number of hydrogens bounded to carbon atoms deduced by comparative analysis of {^1^H}- and APT-^13^C-NMR spectra. Chemical shifts and coupling constants (*J*) obtained of 1D ^1^H-NMR spectrum. Superimposed ^1^H signals are described without multiplicity and approximated chemical shifts deduced by HSQC, HMBC and ^1^H-^1^H-COSY spectra. Assignments deduced by a combination of 1D and 2D (^1^H-^1^H-COSY, HSQC and HMBC) NMR experiments.

## Supplementary Materials

Figures S1–S18 and Schemes S1 and S2 are available online.

## References

[1] Pennington T.D., Styles B.D. (1975). A generic monograph of the Meliaceae. Blumea.

[2] Pupo M.T., Adorno M.A., Vieira P.C., Fernandes J.B., Silva M.F.G.F., Pirani J.R. (2002). Terpenoids and steroids from *Trichilia* species. J. Braz. Chem. Soc..

[3] Rodrigues V.F., Carmo H.M., Braz-Filho R., Mathias L., Vieira I.J.C. (2010). Two new terpenoids from *Trichilia quadrijuga* (Meliaceae). Nat. Prod. Comm..

[4] Vieira I.J.C., Figueiredo E.R., Freitas V.R., Mathias L., Braz-Filho R., Araújo R.M. (2010). A new sesquiterpene from *Trichilia casaretti* (Meliaceae). Am. J. Anal. Chem..

[5] Freitas V.R., Carmo H.M., Oliveira R.R., Braz-Filho R., Mathias L., Vieira I.J.C. (2009). Isolation of terpenoids from *Trichilia quadrijuga* (Meliaceae) by droplet counter-current chromatography. Chromatographia.

[6] Ramírez M.C., Toscano R.A., Arnason J., Omar S., Cerda-Garcia-Rojas C.M., Mata R. (2000). Structure, conformation and absolute configuration of new antifeedant dolabellanes from *Trichilia trifolia*. Tetrahedron.

[7] Xie Y.S., Isman M.B., Gunning P., Mackinnon S., Arnason J.T., Taylor D.R., Sánchez P., Hasbun C., Towers G.H.N. (1994). Biological activity of extracts of *Trichilia* species and the limonoid hirtin against lepidopteran larvae. Biochem. Syst. Ecol..

[8] Cortez D.A.G., Fernandes J.B., Vieira P.C., Silva M.F.G.F., Ferreira A.G., Cass Q.B., Pirani J.R. (1998). Meliacin butenolides from *Trichilia estipulate*. Phytochemistry.

[9] Lago J.H.G., Roque N.F. (2002). Cycloartane triterpenoids from *Guarea macrophylla*. Phytochemistry.

[10] Yang A., Shang Q., Yang L., Li C., Yuan H.J. (2017). Chemical Constituents of the Flowerbuds of *Tussilago farfara*. Chem. Nat. Compd..

[11] Khan M.T.H., Khan S.B., Ather A. (2006). Tyrosinase inhibitory cycloartane type triterpenoids from the methanol extract of the whole plant of *Amberboa ramosa* Jafri and their structure–activity relationship. Bioorg. Med. Chem..

[12] Bohlmann F., Misra L.N., Jakupovic J., King R.M., Robinson H. (1985). Guaianolides, heliangolides, diterpenes and cycloartenol derivatives from *Balsamorhiza sagittata*. Phytochemistry.

