# Supplementary material for: Two Novel Cycloartane-Type Triterpenes from Trichilia casaretti C. DC. (Meliaceae)
Source: Molecules. 2018 Apr 19;23(4):949. doi: 10.3390/molecules23040949 (PMC6017113; doi:10.3390/molecules23040949)
Supplement: Supplementary file 1 [file molecules-23-00949-s001.pdf]

SUPPLEMENTARY MATERIALS

Two Novel Cycloartane-Type Triterpenes from *Trichilia casaretti* C. DC. (Meliaceae)

Ivo J. Curcino Vieira[1*], Elaine R. Figueiredo[2], Milena G. Curcino Vieira[3], Almir R. de Carvalho Junior[1], Michel de Souza Passos[1], Samyra I. da Silva Boeno[1], Otoniel A. Azevedo[4] and Raimundo Braz-Filho[1,5]

[1] Setor de Química de Produtos Naturais, Universidade Estadual do Norte Fluminense Darcy Ribeiro, 28013-602, Campos dos Goytacazes, Rio de Janeiro, Brazil.

[2] Instituto Federal Fluminense, *Campus* Bom Jesus do Itabapoana, Avenida Dario Vieira Borges, 235, Parque do Trevo, 28360-000, Bom Jesus do Itabapoana, Rio de Janeiro, Brazil.

[3] Faculdade de Medicina de Campos, Avenida Alberto Torres, 217, Centro Campos dos Goytacazes, 28035-581, Rio de Janeiro, Brazil.

[4] Centro Universitário São Camilo, Campus I, Rua São Camilo de Léllis 01, 29304-910 Cachoeiro de Itapemirim , Espirito Santo, Brazil.

[5] Departamento de Química, Universidade Federal Rural do Rio de Janeiro, CP 74541, 23890-000 Seropédica, Rio de Janeiro, Brazil.

*Correspondence: e-mail:curcino@uenf.br; Tel: + 55-22-27486504

Received: date; Accepted: date; Published: date

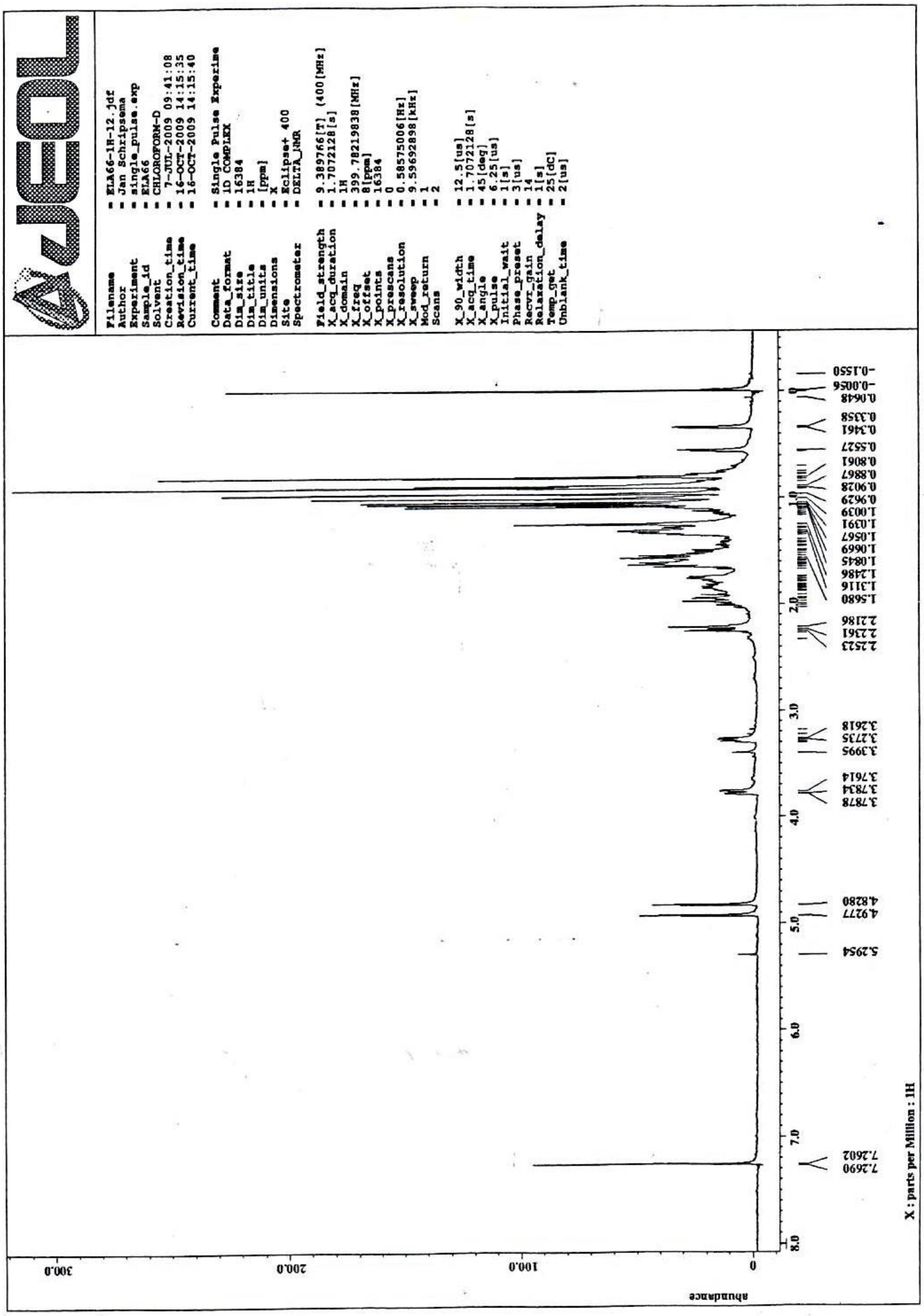

Figure S1. ^{1}H NMR spectrum of compound **1** (400 MHz, $CDCl_3$).

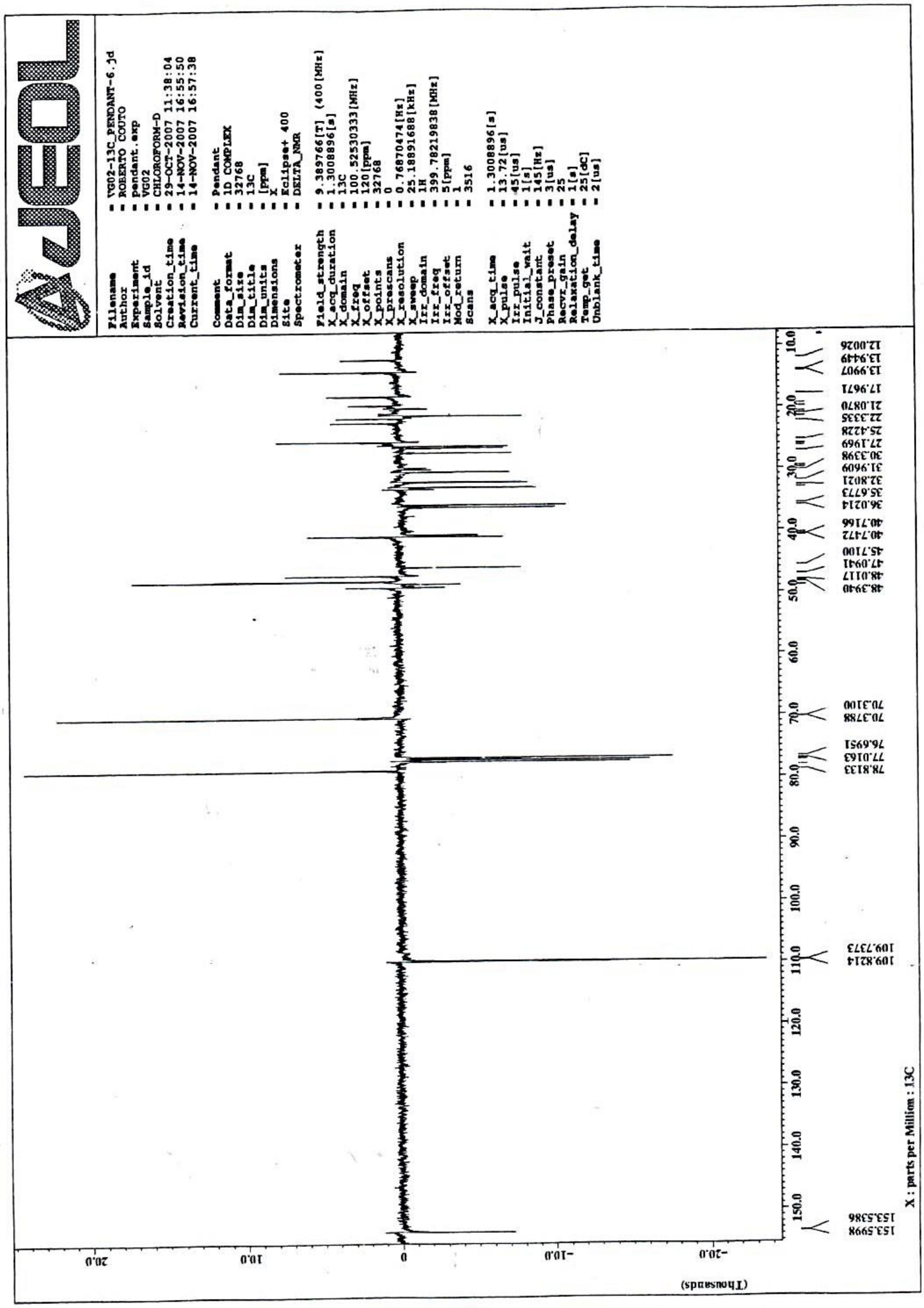

Figure S2. ^{13}C NMR spectrum of compound **1** (100 MHz, $CDCl_3$).

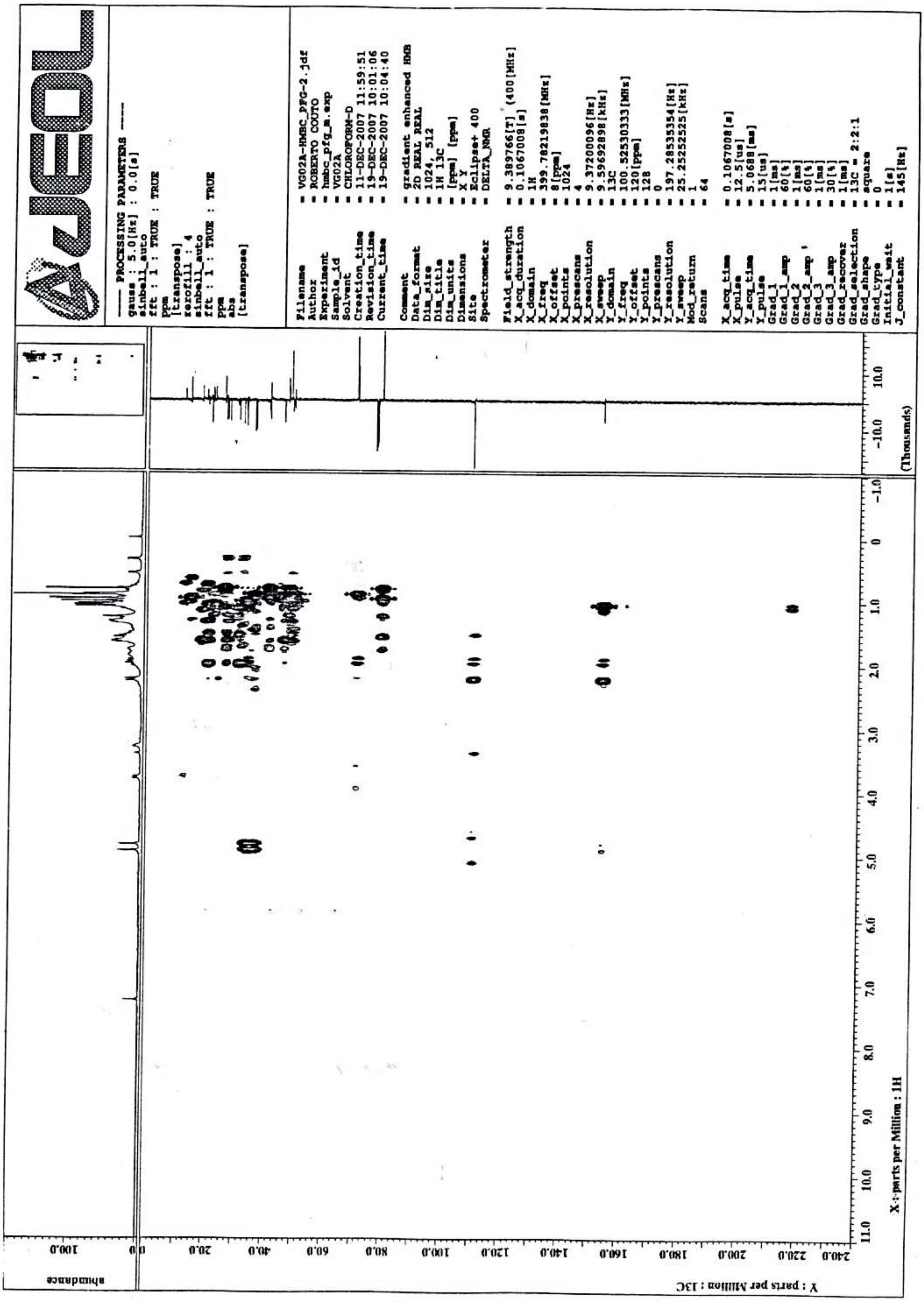

Figure S3. HMBC spectrum of compound **1** ($CDCl_3$).

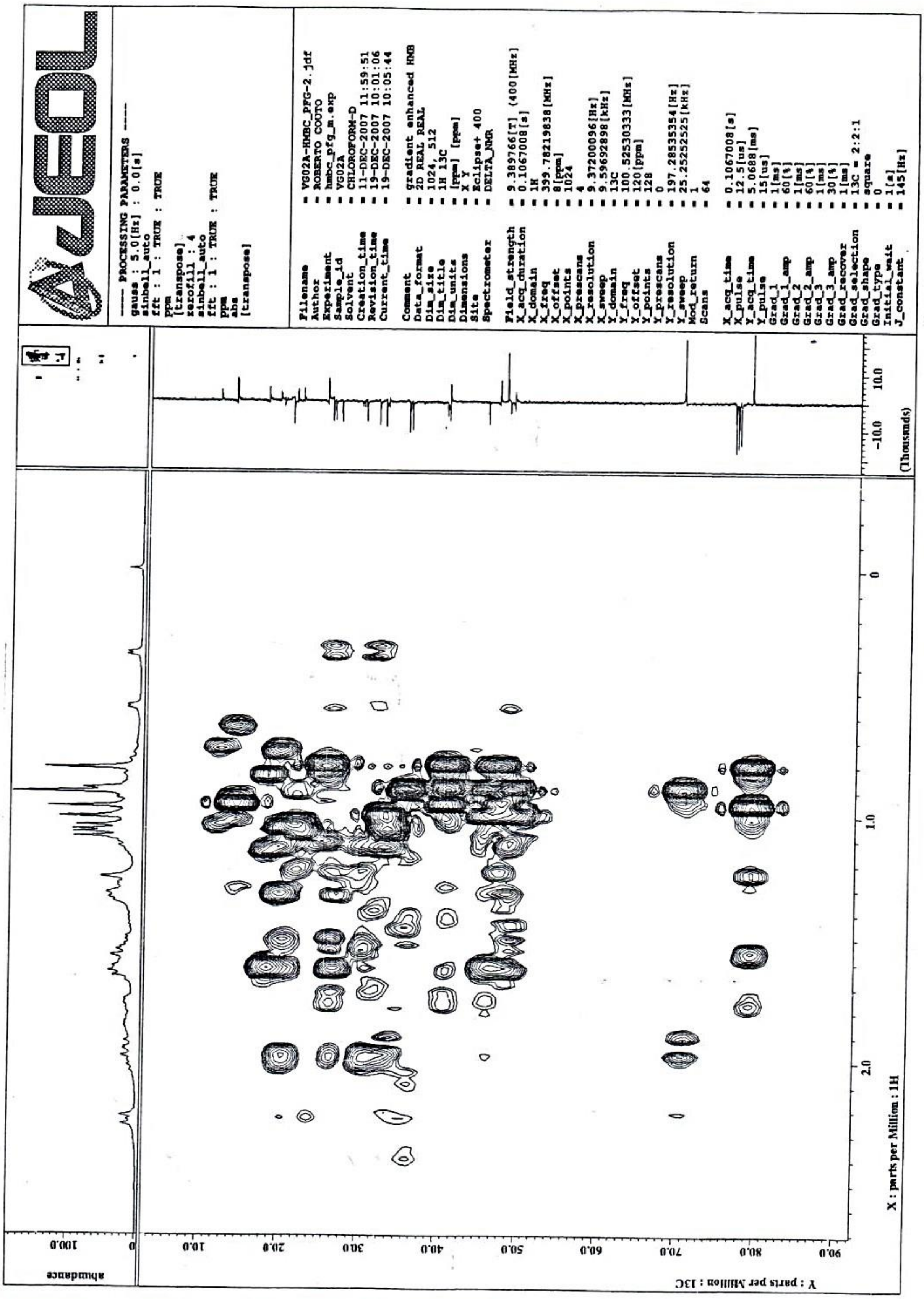

Figure S4. Extension of HMBC spectrum of compound **1** ($CDCl_3$).

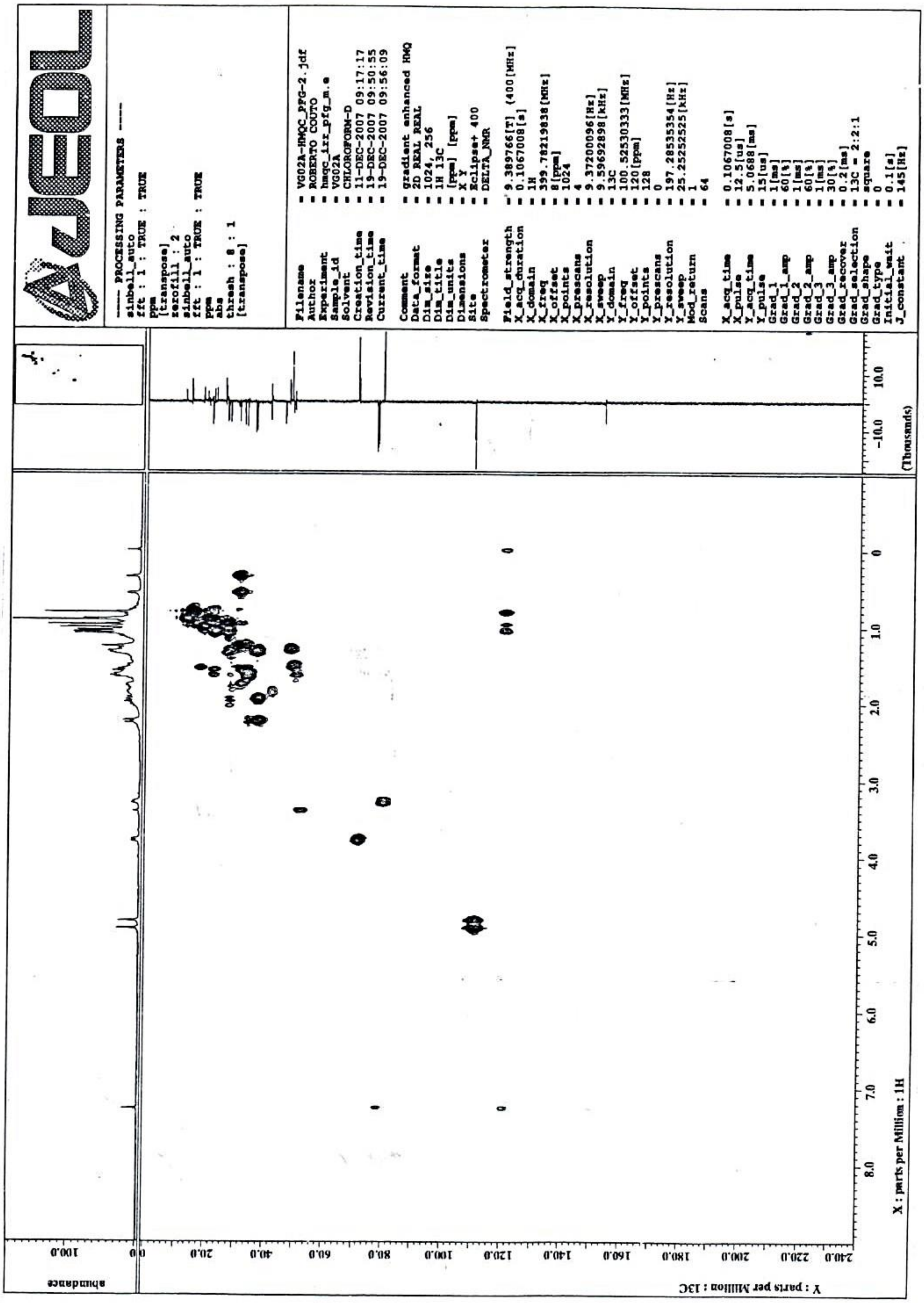

Figure S5. HMQC spectrum of compound **1** ($CDCl_3$).

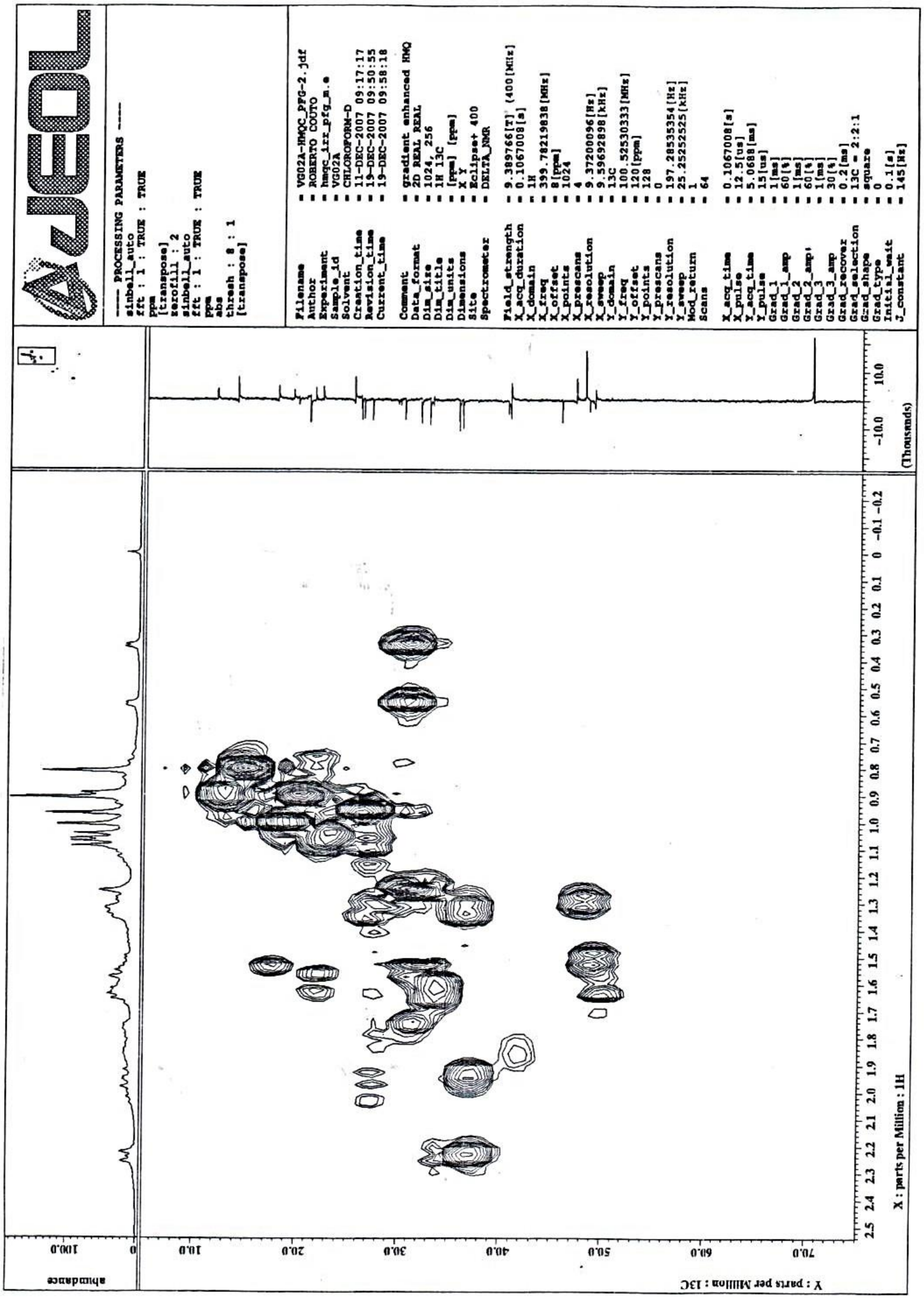

Figure S6. Extension of HMQC spectrum of compound **1** ($CDCl_3$).

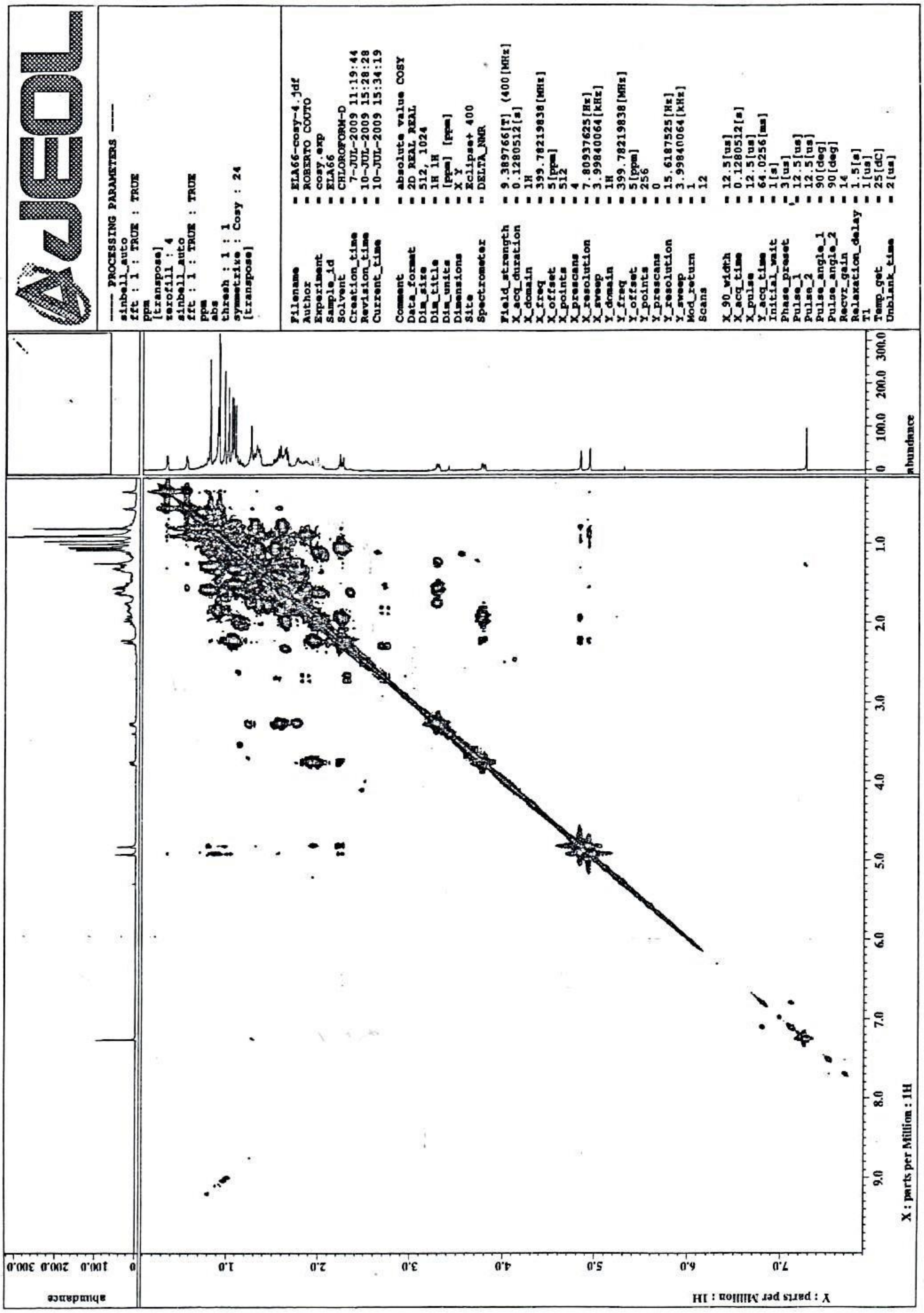

Figure S7. ^{1}H-^{1}H-COSY spectrum of compound **1** (400 MHz, $CDCl_3$).

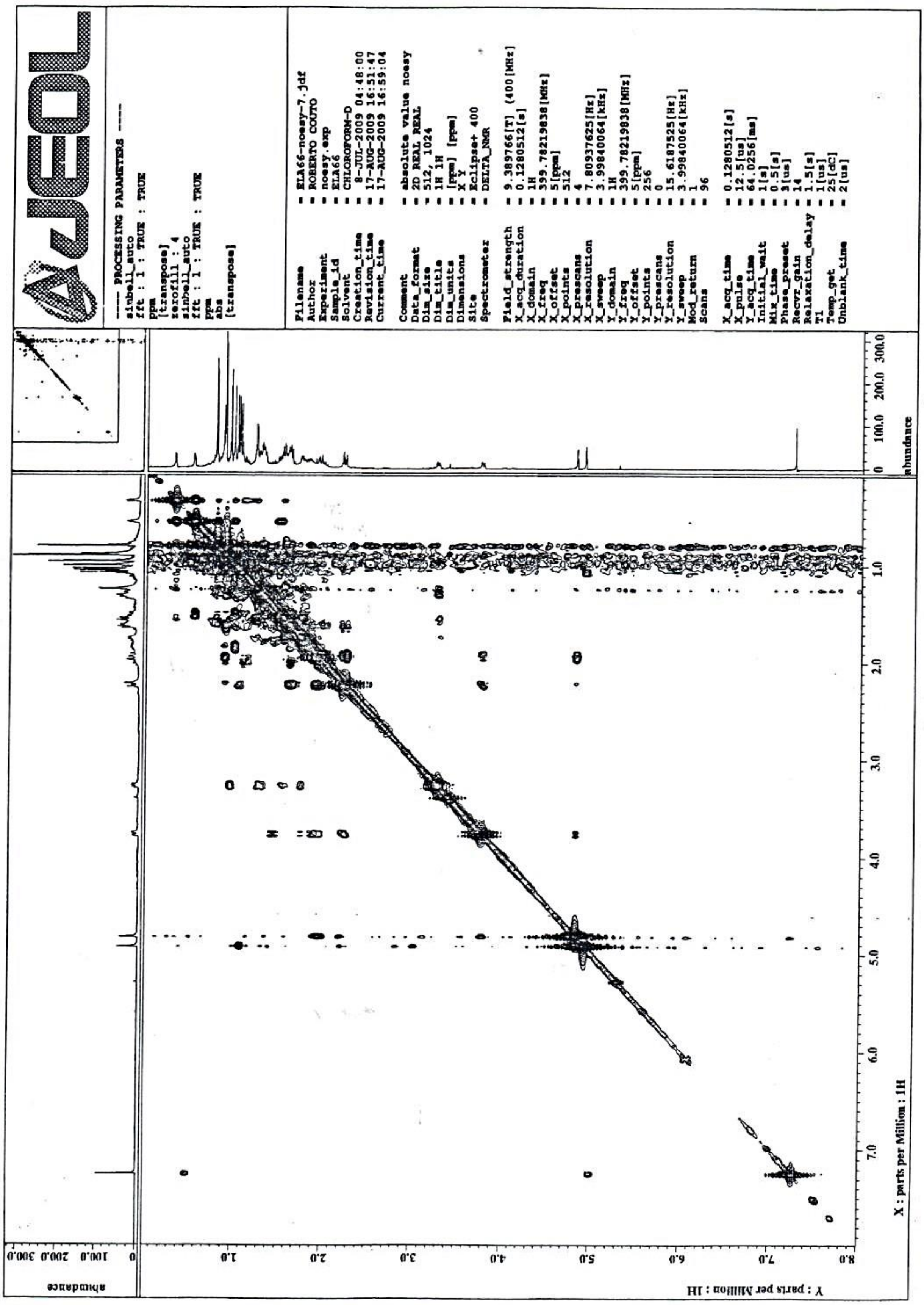

Figure S8. ^{1}H-^{1}H-NOESY spectrum of compound **1** (400 MHz, $CDCl_3$).

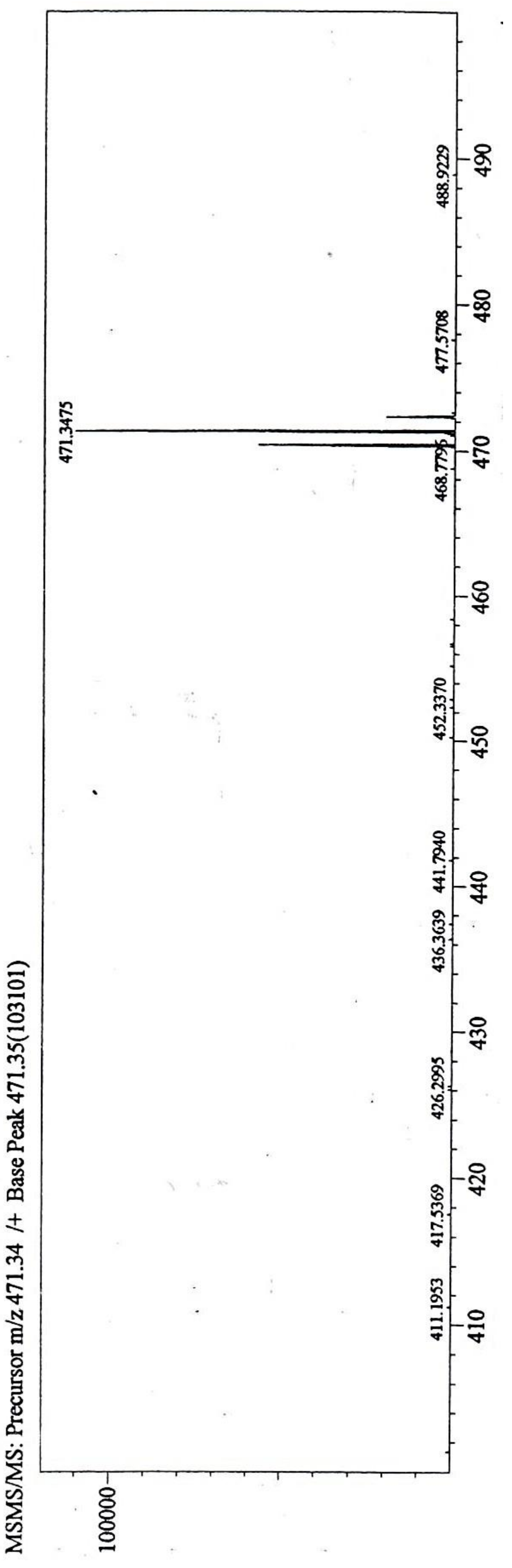

Figure S9. HR-ESI/MS spectrum of compound **1**.

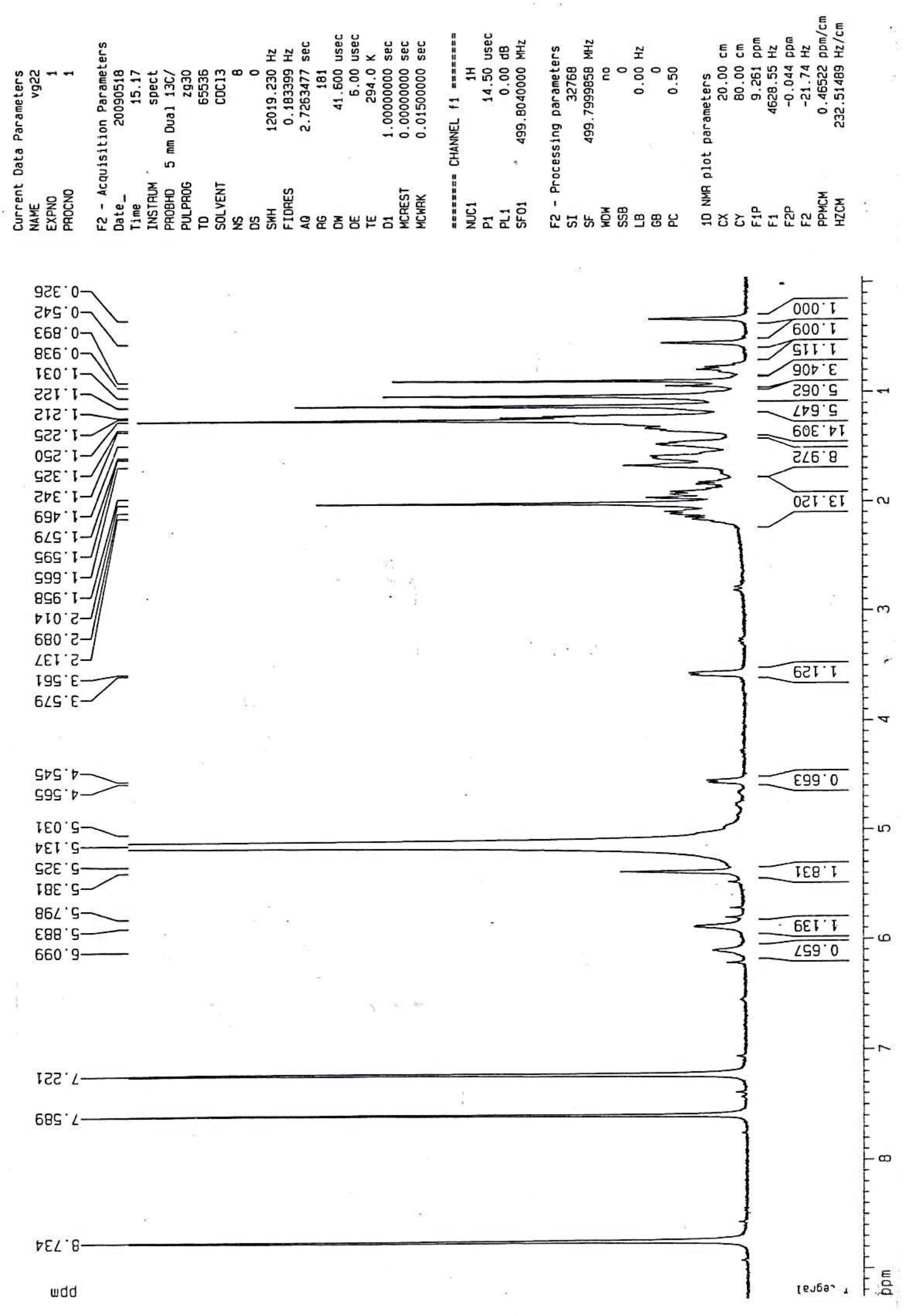

Figure S10. ^{1}H NMR spectrum of compound **2** (500 MHz, Pyridine-d_5).

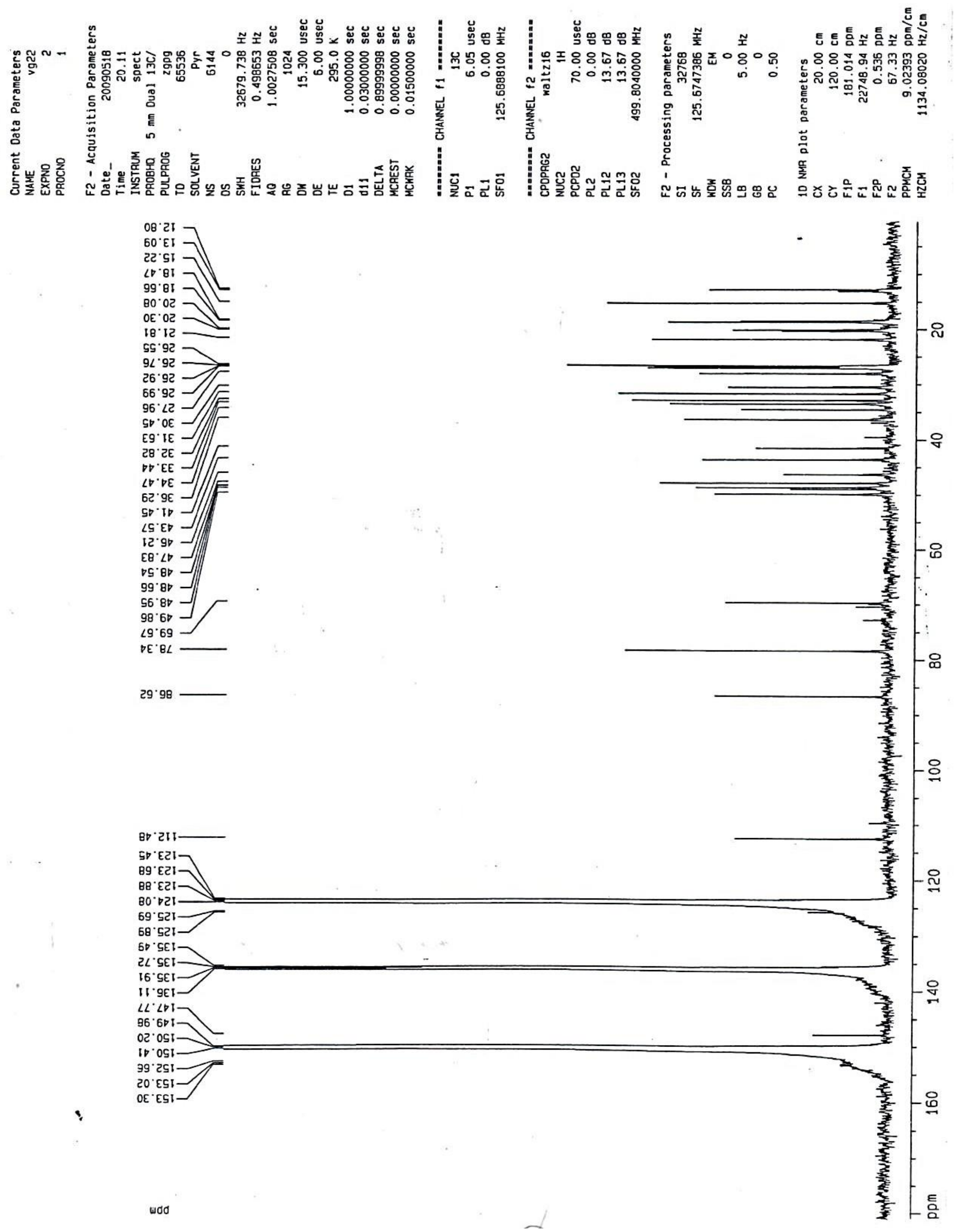

Figure S11. ^{13}C NMR spectrum of compound **2** (125 MHz, Pyridine-d_5).

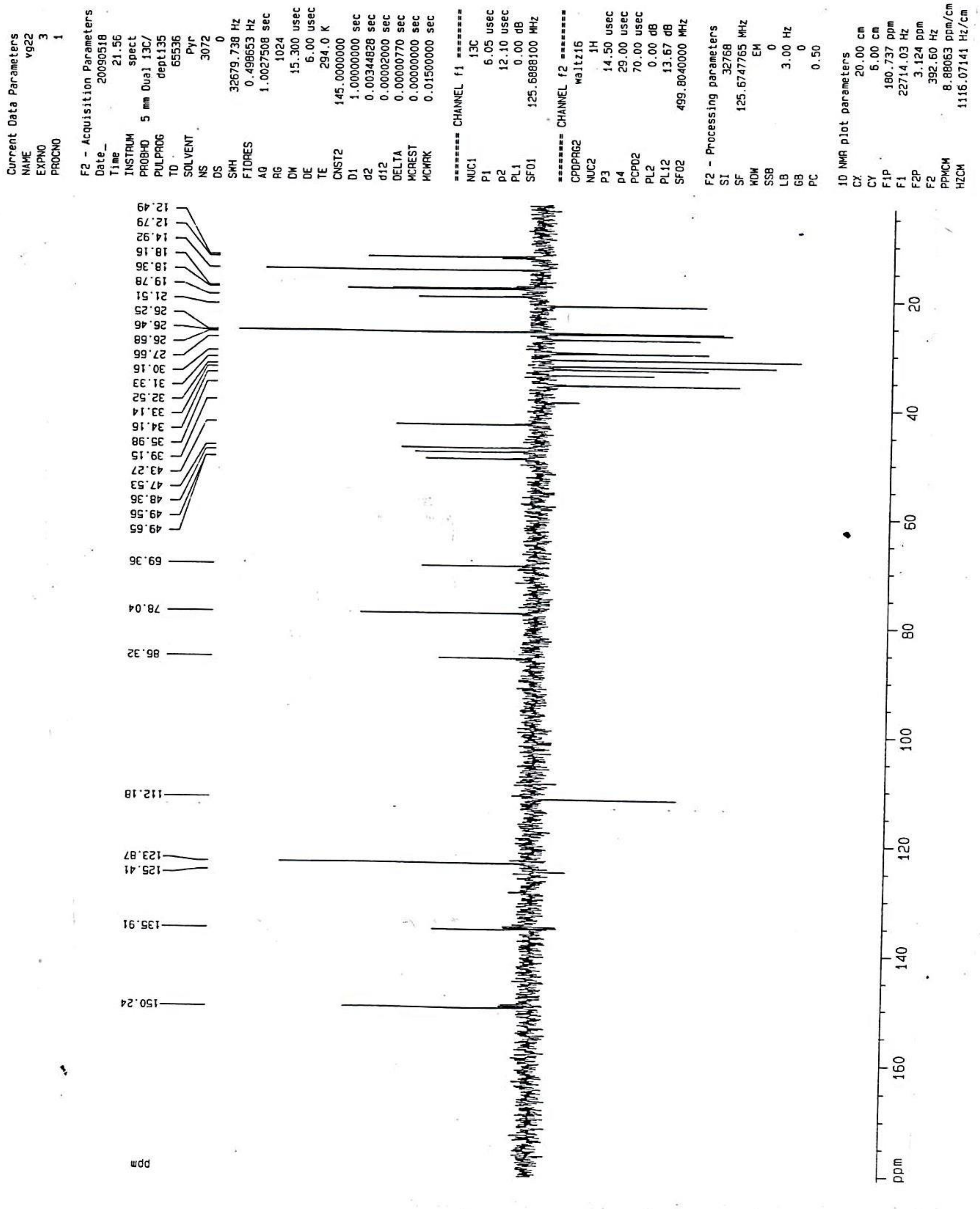

Figure 12. ^{13}C NMR-APT spectrum of compound **2** (125 MHz, Pyridine-d_5).

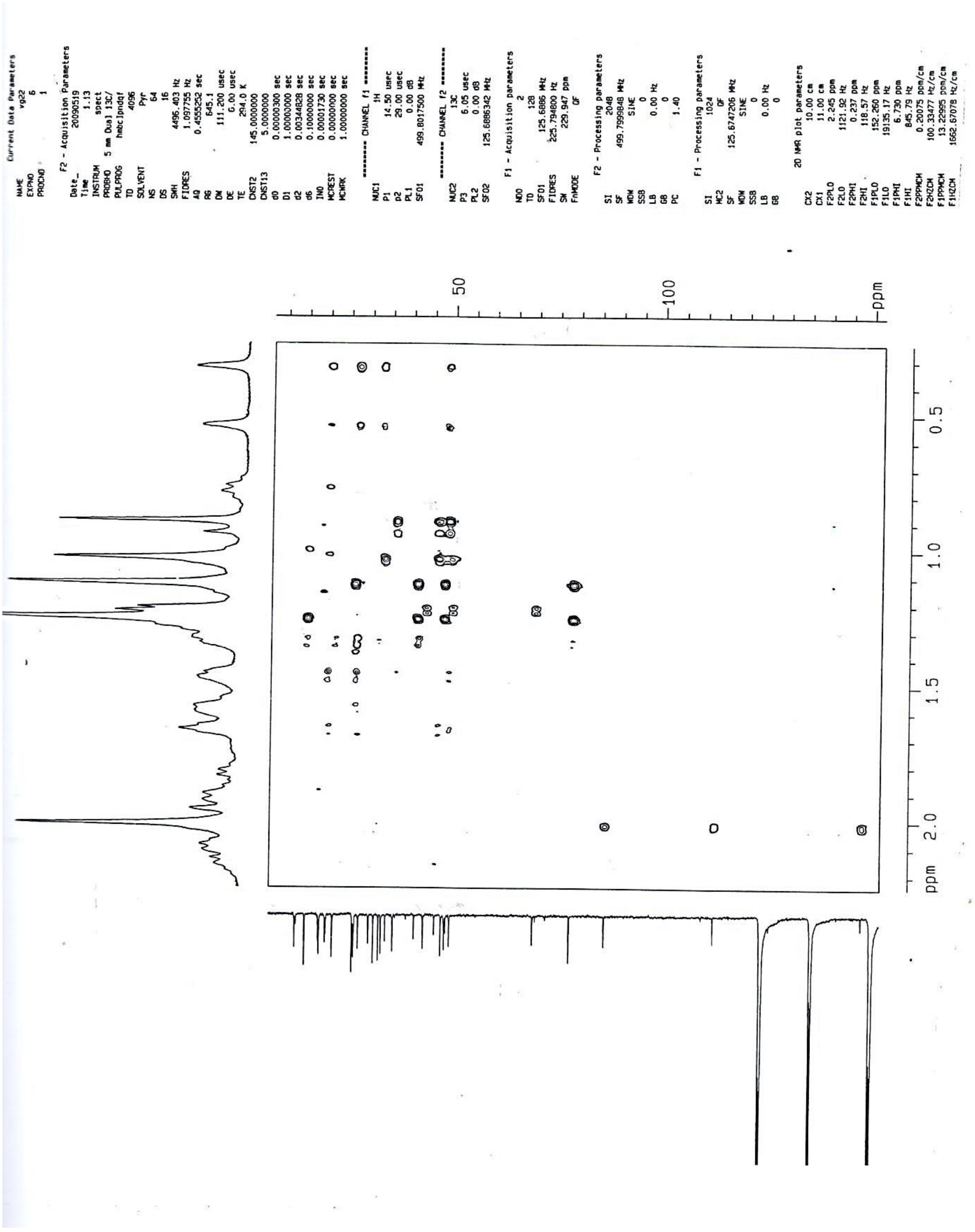

Figure S13. HMBC spectrum of compound **2** (Pyridine-*d*$_5$).

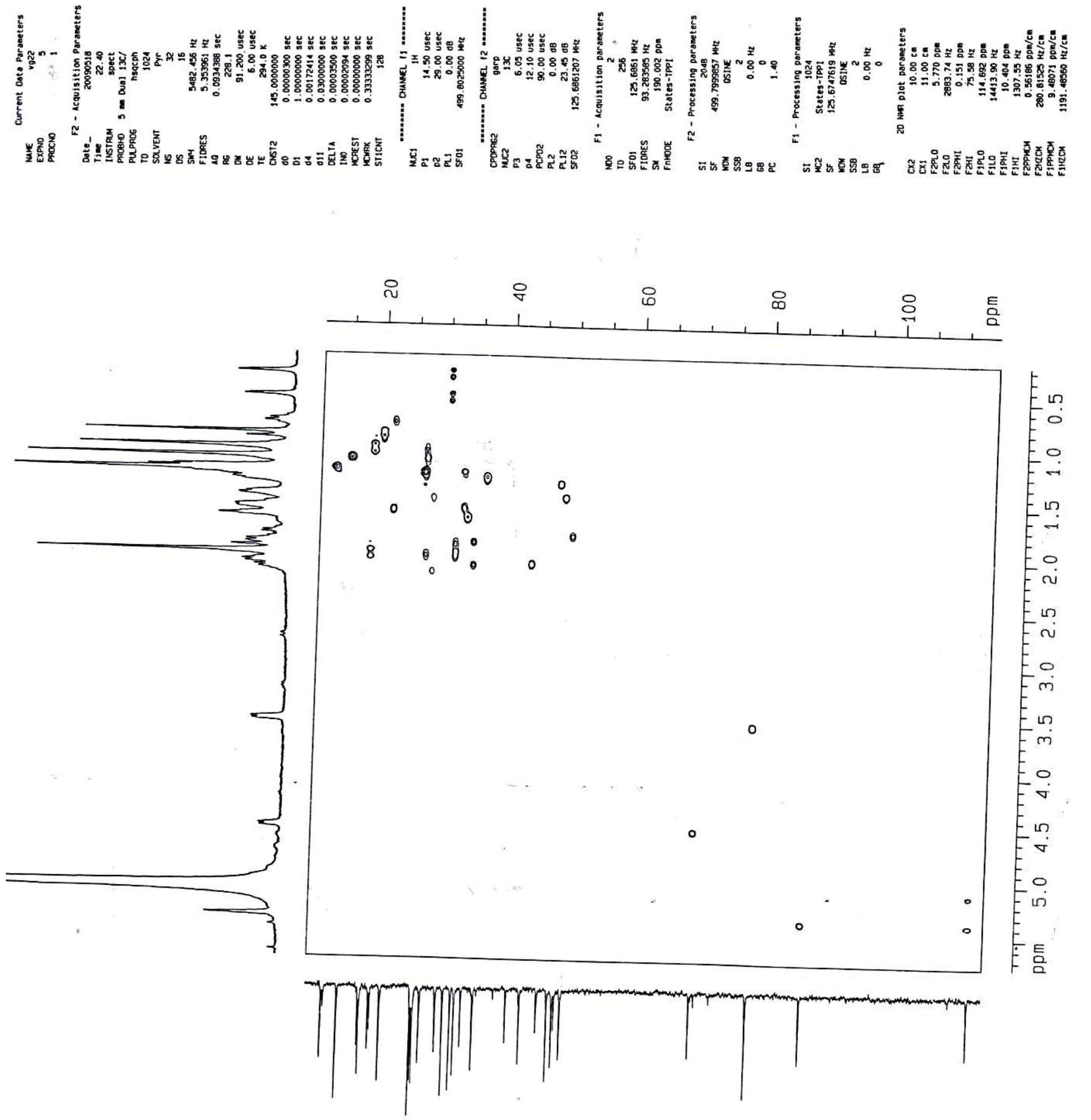

Figure S14. HMQC spectrum of compound **2** (Pyridine-d_5).

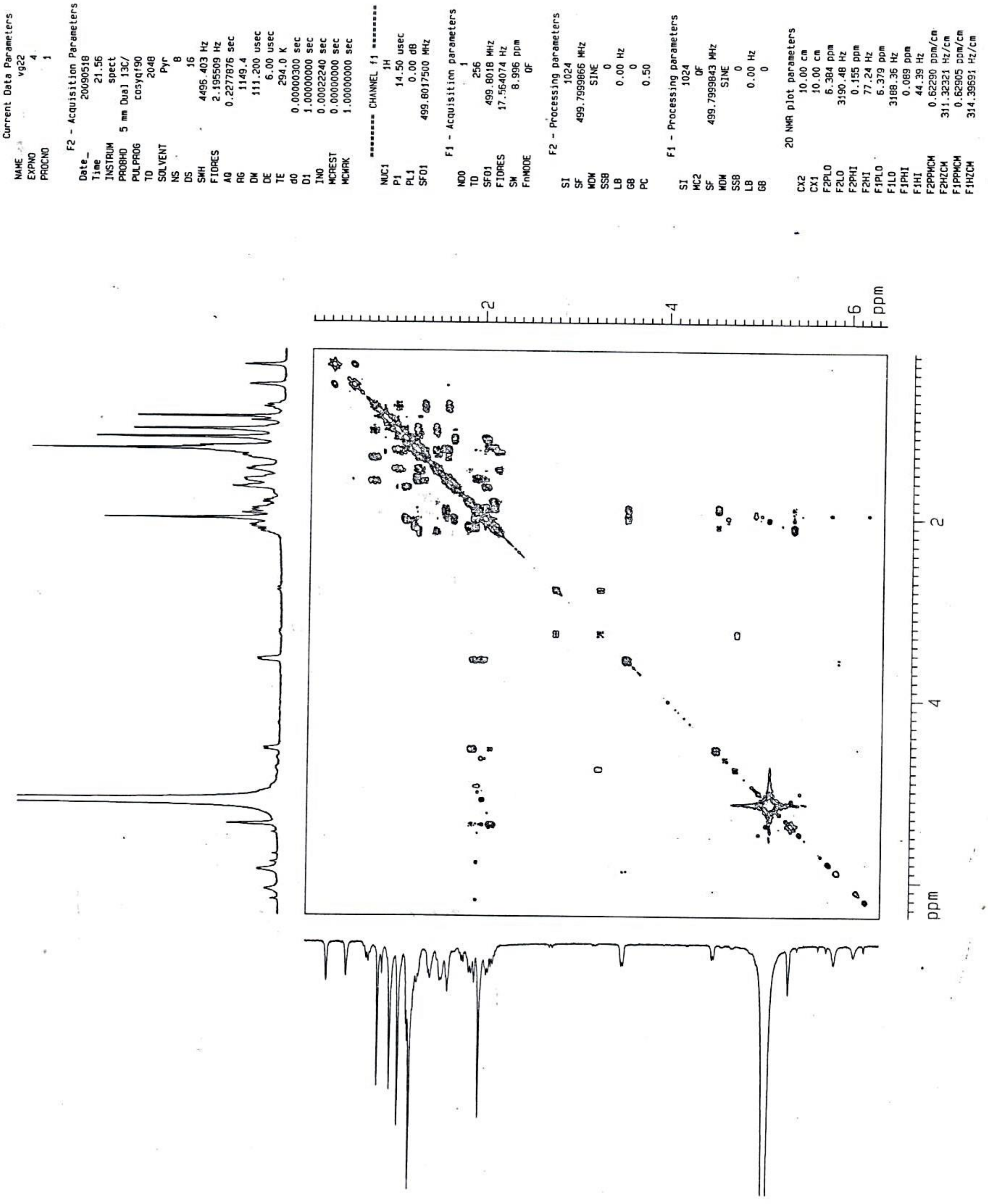

Figure S15. ^{1}H-^{1}H-COSY spectrum of compound **2** (Pyridine-d_5).

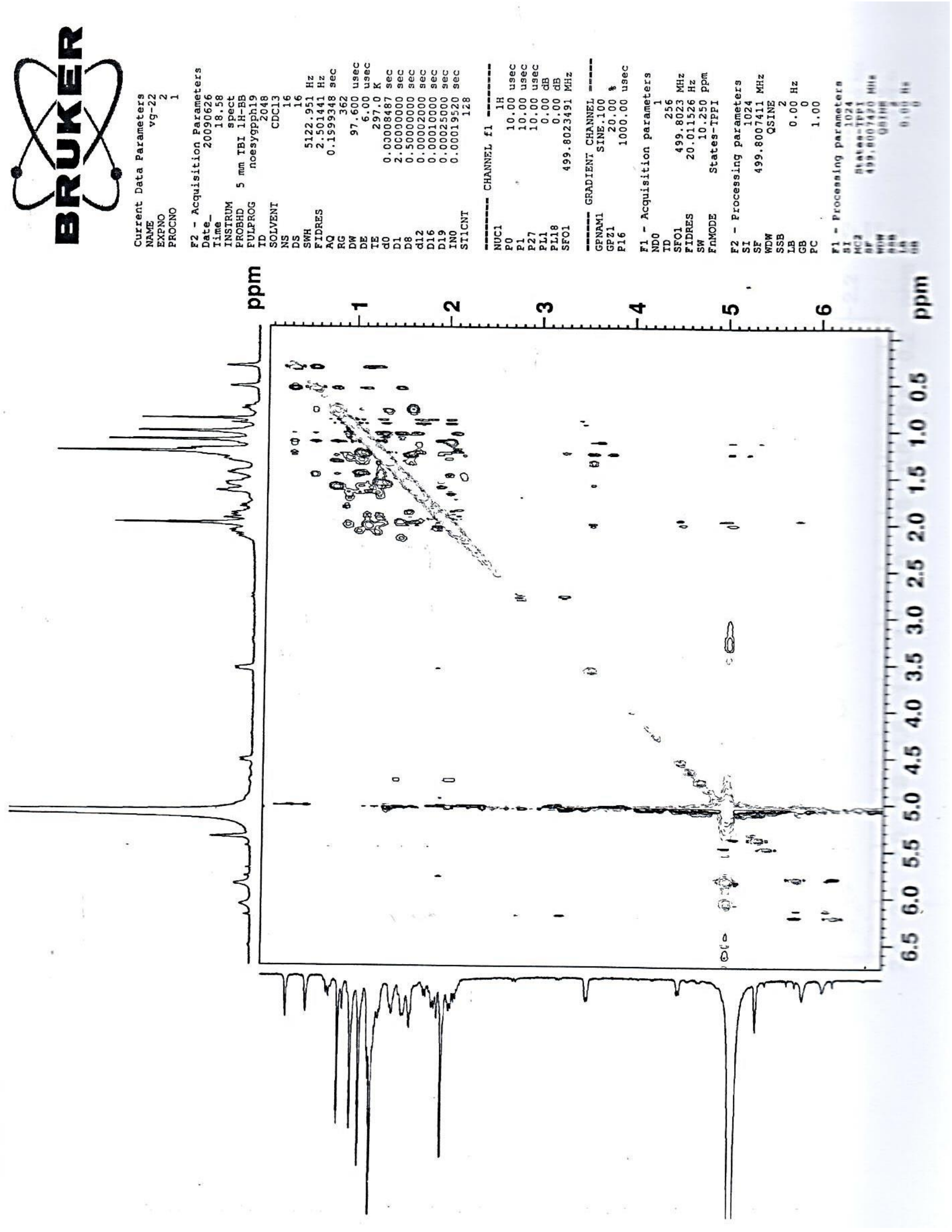

Figure S16. ^{1}H-^{1}H-NOESY spectrum of compound **2** (Pyridine-d_5).

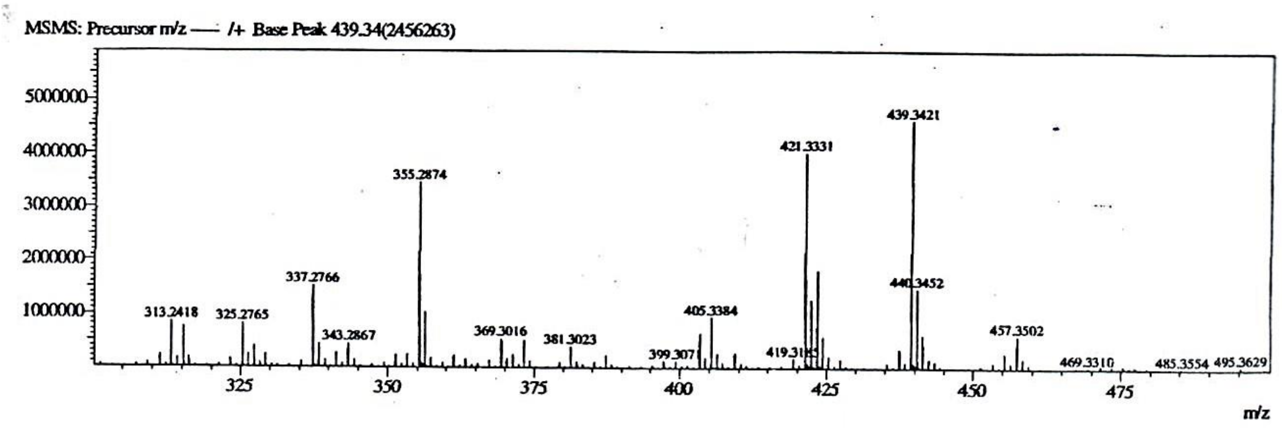

Figure S17. HR-ESI/MS spectrum of compound **2**.

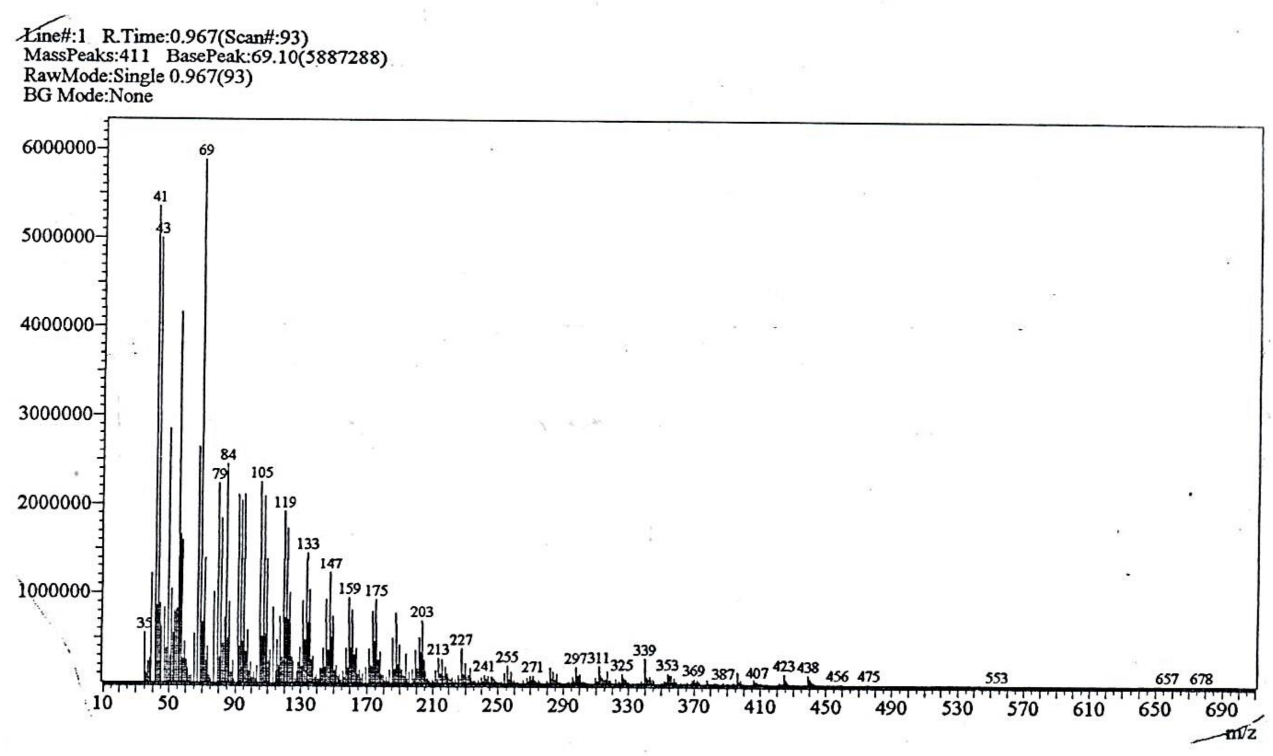

Figure S18. LR-ESI/MS spectrum of compound **2**.

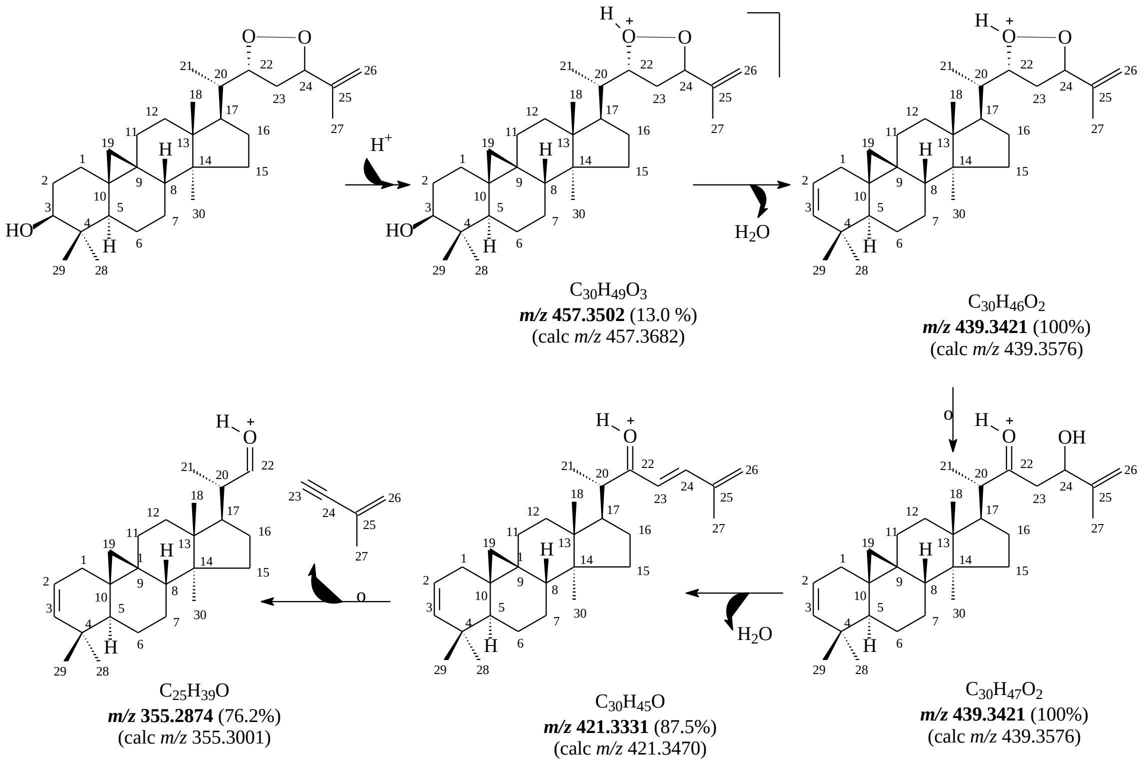

Scheme S1. Fragments proposed to justify the main peaks observed in the HRESIMS of **2**.

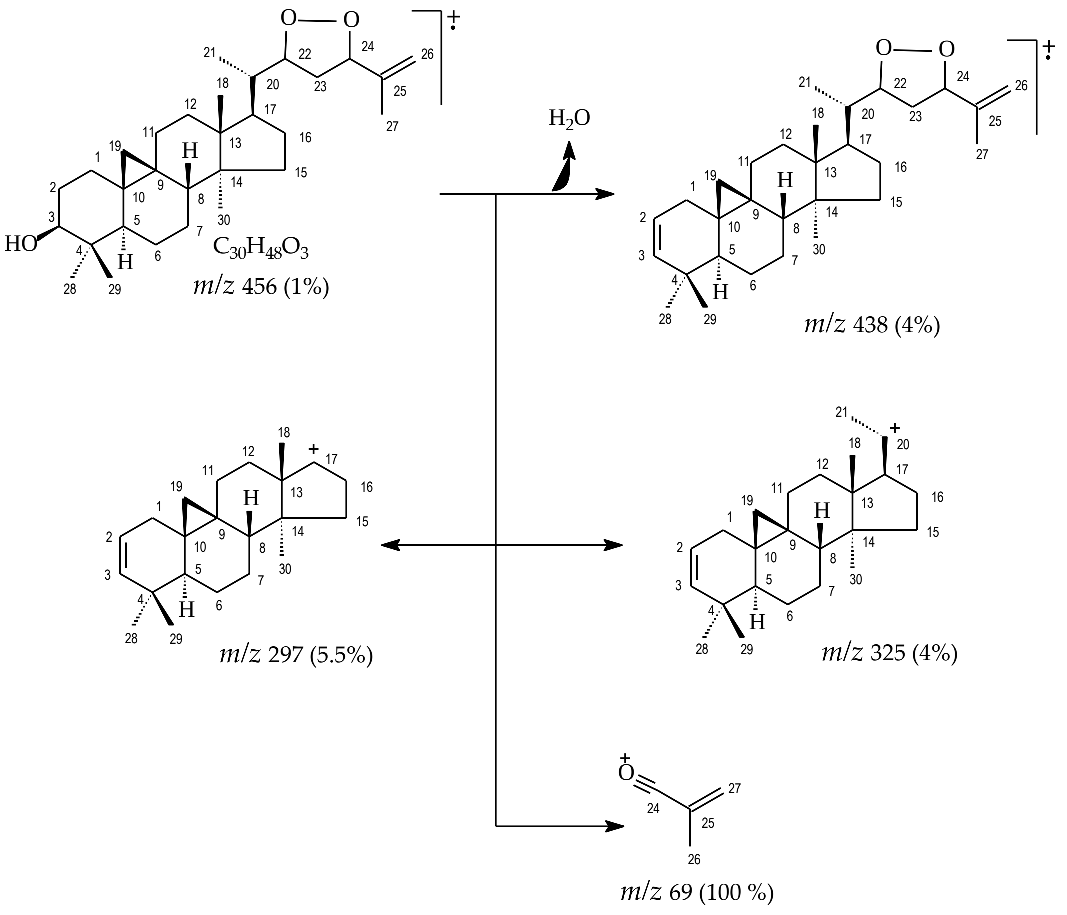

Scheme S2. Fragments proposed to justify the main peaks observed in the EIMS (70 eV) of **2**.
